# Supplementary figures and images for: Metabolomic Analysis Reveals the Metabolic Diversity of Wild and Cultivated Stellaria Radix (Stellaria dichotoma L. var. lanceolata Bge.)
Source: Plants (Basel). 2023 Feb 9;12(4):775. doi: 10.3390/plants12040775 (PMC9959334; doi:10.3390/plants12040775)

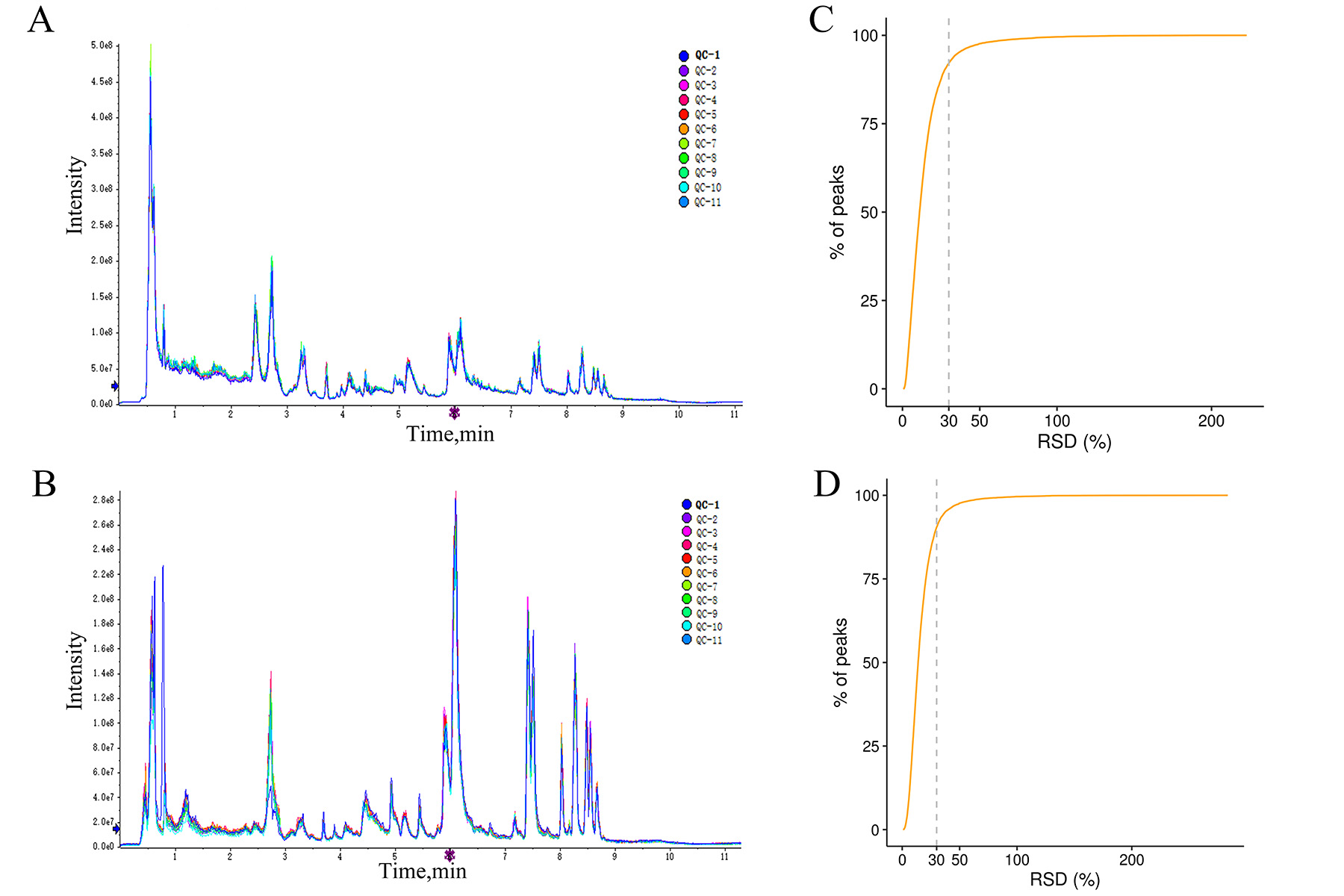

Supplement: Supplementary file 1 [file plants-12-00775-s001.zip › plants-2091280-Supplementary materials/Supplementary Figure S1. Quality control analysis of YCH metabolites detection..jpg]
